# CLDN8 and ABCA12 define a shared molecular signature in ulcerative colitis-psoriasis comorbidity

**DOI:** 10.3389/fimmu.2026.1862149

**Published:** 2026-08-05

**Authors:** Han Wang, Kun Jin, Yuan Zhao, Ying Ruan, Huiqin Zhu, Ni Zhu

**Affiliations:** 1 School of Pharmacy, Xianning Medical College, Hubei University of Science and Technology, Xianning, China; 2 Hubei Key Laboratory of Environmental Risks and Related Diseases Precision Control, School of Basic Medicine Sciences, Xianning Medical College, Hubei University of Science and Technology, Xianning, China; 3 School of Biomedical Engineering, Xianning Medical College, Hubei University of Science and Technology, Xianning, China; 4 National Demonstration Center for Experimental General Medicine Education, Xianning Medical College, Hubei University of Science and Technology, Xianning, China

**Keywords:** ABCA12, CLDN8, machine learning, psoriasis, ulcerative colitis

## Abstract

**Background:**

Ulcerative colitis (UC) and psoriasis (PsO) are two common immune-mediated inflammatory diseases with significant comorbidity, yet whether one directly causes the other or they share common genetic and immunopathological backgrounds remains unclear. This study integrated bioinformatics, animal experiments, and bidirectional Mendelian randomization to systematically identify potential common molecular targets mediating the comorbidity.

**Methods:**

Transcriptomic data for UC and PsO were obtained from the Gene Expression Omnibus (GEO) database. Differential expression genes (DEGs) and WGCNA were performed to identify common transcriptomic alterations. Then, machine learning algorithms (SVM, RF, XGBoost, GLM) were used to screen for shared signature genes. The nomogram diagnostic models were constructed, and immune infiltration landscape were analyzed. An acute UC-PsO comorbidity mouse model was established by simultaneous administration of dextran sulfate sodium and imiquimod, and the expression levels of key genes were examined. Finally, bidirectional Mendelian randomization (MR) was performed to assess the genetic causal relationship between UC and PsO.

**Results:**

3,840 DEGs in UC and 4,208 in PsO, with 619 overlapping DEGs, were identified. WGCNA further screened 17 up−regulated and 16 down−regulated co−expressed genes common to both diseases. Cross−validation using four machine learning algorithms finally identified CLDN8 and ABCA12 as the core signature genes shared by UC and PsO. Diagnostic models based on these two genes performed well for both diseases (specific AUC values can be added). Immune infiltration analysis revealed similar inflammatory pathway characteristics between the two diseases. In the UC−PsO comorbidity animal model, the expression changes of CLDN8 and ABCA12 were consistent with those in clinical samples, confirming their functional relevance. Bidirectional MR analysis showed no significant causal effect of UC on PsO nor of PsO on UC, which does not support the direct causation hypothesis but instead supports the shared mechanism hypothesis.

**Conclusion:**

CLDN8 and ABCA12 shared molecular signature in UC-PsO comorbidity, however, no direct genetic causal relationship between UC and PsO. Clinically, each disease should be managed according to its own pathology, while targeting common inflammatory pathways involving CLDN8/ABCA12 to enable personalized treatment.

## 1 Introduction

Inflammatory Bowel Disease (IBD) encompasses a spectrum of chronic, relapsing intestinal inflammatory disorders, with Ulcerative Colitis (UC) and Crohn’s Disease constituting its principal forms. As the predominant IBD subtype, UC is characterized by persistent inflammation confined to the colon and rectum, yet its etiopathogenesis remains incompletely elucidated; current evidence implicates a multifactorial interplay involving genetic susceptibility, environmental triggers, gut microbial dysbiosis, and dysregulated mucosal immune responses (1). The global burden of UC continues to escalate, as reflected by steadily rising incidence and prevalence rates—a trend particularly pronounced in industrialized nations but increasingly evident in newly industrialized and developing regions as well (2). Patients with UC not only have to endure gastrointestinal symptoms such as diarrhea and blood in stool over a long period but often also experience multi-system manifestations including joint, skin, and eye issues, severely threatening their quality of life and leading to significant medical costs (3). Despite the continuous enrichment of treatment options for UC over the past two decades, which comprises 5-aminosalicylic acid derivatives, corticosteroids, immunomodulators, and biological agents, 10-20% of patients still respond poorly to existing medications and ultimately require surgical treatment (4). These clinical challenges indicate that a deeper exploration of the pathogenesis of UC and its relationship with other chronic inflammatory diseases has become an urgent research need.

As a systemic inflammatory condition, UC extends its influence beyond the gut, exhibiting a high burden of extraintestinal comorbidities. Among these, the link with psoriasis (PsO) has garnered particular attention. PsO is a chronic inflammatory dermatosis driven by dysregulated keratinocyte proliferation and infiltration of immune cells, pathophysiological features that strikingly parallel the epithelial barrier dysfunction seen in UC. UC and PsO, as immune-mediated chronic inflammatory diseases, constitute a significant public health burden worldwide. Epidemiological evidence indicates that UC occurs in 0.5% of individuals with PsO (5), while the reciprocal observation reveals that PsO affects approximately 2.8% of patients diagnosed with UC (5). Large-scale population studies further reveal a significant bidirectional association between the two diseases: the risk of UC in patients with PsO is 1.71 (95% CI: 1.55-1.89) (6), while the odds ratio of PsO in patients with UC is 1.75 (95% CI: 1.49-2.05) (6). Notably, this relationship exhibits greater magnitude in younger populations; specifically, individuals under 19 years of age with PsO demonstrate a hazard ratio for developing inflammatory bowel disease of 5.33 [95% confidence interval (CI): 3.74–7.59] (7). This phenomenon has been corroborated by regional investigations spanning diverse ethnic cohorts. For instance, a Korean population-based analysis revealed that a diagnosis of PsO confers an elevated subsequent risk of UC, with an adjusted odds ratio calculated at 1.77 (95% CI: 1.44–2.18) (8). Furthermore, evidence from a 20-year nationwide Danish cohort study indicates that the incidence of UC is heightened among PsO patients undergoing various therapeutic regimens (9). These comorbid relationships may even begin to manifest up to ten years before diagnosis (10), suggesting that the two diseases may share underlying pathophysiological mechanisms. The above epidemiological evidence highlights the public health significance of UC and PsO and provides a solid context for future research into the potential associations between them. A Danish nationwide cohort investigation has documented a bidirectional relationship between UC and PsO, demonstrating not only an elevated incidence of PsO among individuals with UC but also a substantially heightened susceptibility to UC in the PsO population (risk increased by 49% in patients with mild PsO and by 56-96% in patients with severe PsO) (11). Systematic reviews and meta-analyses further quantified this association, showing a 71% increased risk of UC in patients with PsO (6). A nationwide cohort study in Korea also confirmed a significant association between PsO and increased risk of UC (adjusted odds ratio 1.77) (8). Collectively, these findings underscore a robust link and a notable propensity for comorbid occurrence between UC and PsO.

PsO is a disease characterized by chronic, immune-mediated skin inflammation, with a global prevalence of approximately 2%. It not only affects the skin but is also associated with multi-system diseases such as arthritis, cardiovascular issues, and metabolic disorders (12). Patients with PsO face long-term skin damage, itching, and psychological stress, significantly impairing their quality of life (13). Recent advances in molecularly targeted therapies and biologic agents have substantially improved the therapeutic landscape for PsO; nonetheless, some patients still experience limited efficacy and drug-related adverse reactions (14, 15). Importantly, epidemiological and clinical observations show that PsO co-occurs with various autoimmune diseases, including IBD, particularly with a higher comorbidity rate of PsO and UC compared to the general population (16). Both conditions show certain overlaps in pathogenesis, genetic susceptibility, and expression of inflammatory factors, suggesting a potential common pathological basis (17). However, despite existing research focusing on the epidemiological association and partial molecular mechanisms between UC and PsO, the specific molecular basis underlying their comorbidity and its clinical significance still urgently require systematic elucidation (18). Current studies are mostly concentrated on the pathogenesis and treatment of UC or PsO as single diseases, with limited basic research on their comorbidity. Some genomic and transcriptomic analyses have shown intersections between the two in terms of immune response, inflammatory factors, and signaling pathways, such as abnormal IL-23/Th17 axis and upregulation of chemokines; however, the related results are mostly preliminary findings, lacking a systematic co-expression network and comprehensive multi-angle validation (19). Moreover, research that solely relies on traditional bioinformatics mining is often affected by dataset heterogeneity, insufficient sample size, and limitations of analysis methods, which consequently casts uncertainty upon the robustness and practical clinical applicability of candidate comorbidity genes (20). Therefore, there is an urgent need to integrate multiple datasets and employ multi-level analytical methods to thoroughly screen and validate the core genes and regulatory networks of comorbidity between UC and PsO, providing a solid foundation for revealing the comorbidity mechanism and identifying potential therapeutic targets (21). Against this backdrop, the present investigation centers on elucidating the molecular underpinnings of UC–PsO comorbidity. To overcome the constraints inherent in conventional research paradigms, an integrative analytical framework was adopted, comprising differential expression profiling, Weighted Gene Co-expression Network Analysis (WGCNA), and a consensus strategy incorporating multiple mainstream machine learning algorithms (22, 23). Differential expression analysis can systematically screen for disease-related genes, while WGCNA helps to reconstruct gene regulatory networks and identify disease-related functional modules and key genes (24). Various machine learning algorithms further enhance the robustness and biological significance of the screening results from statistical and predictive perspectives, increasing the clinical application potential of candidate genes (25). The genetic causal nexus between the two disorders was further interrogated using Mendelian randomization. Through cross-validation with multiple methods, we can minimize biases associated with single analyses and improve the accuracy and reliability of identifying key comorbidity genes. This study systematically identified and validated diagnostic genes associated with the comorbidity of UC and PsO through integrated omics analysis and multi-algorithm screening, and explored their roles in immune regulation and inflammatory responses. The findings suggest that CLDN8 and ABCA12 may serve as potential common diagnostic biomarkers for UC and PsO comorbidity, with their involvement in immune cell differentiation and migration potentially linked to the inflammatory processes underlying both diseases. This investigation yields novel perspectives on the molecular pathogenesis underpinning UC–PsO comorbidity and establishes a conceptual framework to inform the future development of precision diagnostic modalities and targeted therapeutic interventions. The integrative approach used here may also inform future studies on comorbid mechanisms in other autoimmune diseases and support the advancement of personalized medicine.

## 2 Materials and methods

### 2.1 Data collection and preprocessing

**Figure 1:**
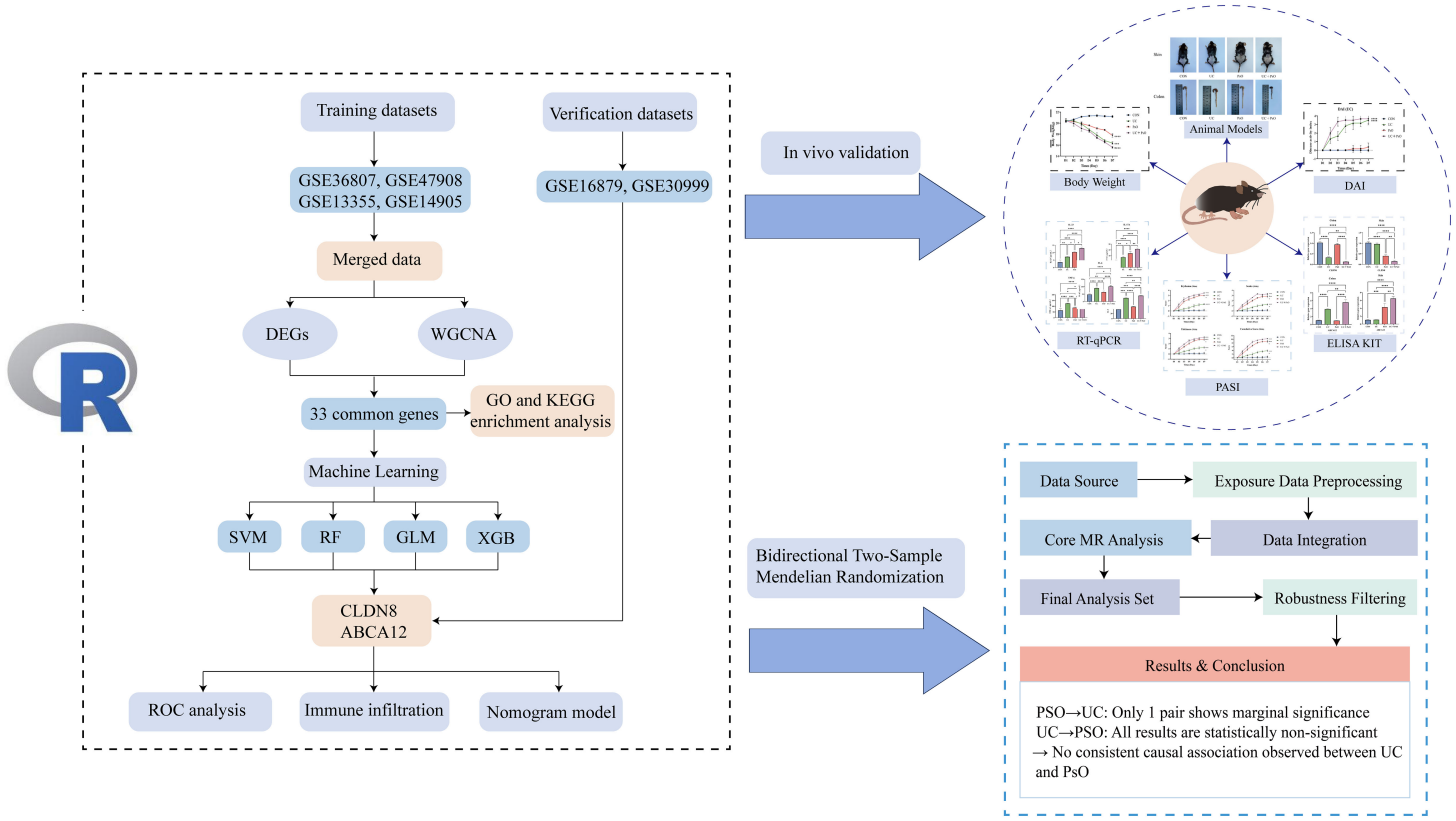
Flowchart of this study.

The flowcharts were shown in **Figure 1**. Transcriptome datasets related to UC and PsO were obtained from the Gene Expression Omnibus (GEO, www.ncbi.nlm.nih.gov/geo). Datasets were selected based on the availability of both disease and healthy control samples, adequate sample size for robust differential expression analysis, unambiguous platform annotation with data amenable to normalization, and complete clinical information—particularly for PsO, where lesional, non-lesional, and control samples were required to be clearly labeled. Datasets generated on incompatible microarray platforms or containing samples with documented pharmacological intervention prior to biopsy collection were excluded. After this rigorous screening, the UC dataset selected includes GSE36807, GSE47908, and GSE16879, which contain normal control samples and UC patient samples; the PsO dataset selected includes GSE13355, GSE14905, and GSE30999, which contain normal control samples and PsO patient samples (**Table 1**). Raw expression data underwent background adjustment and normalization utilizing R software (v4.3.2). Inter-batch technical variation was mitigated by applying the normalizeBetweenArrays routine implemented within the “limma” package. Subsequent quality assessment was conducted via principal component analysis (PCA), and outlier samples were removed to ensure the reliability of subsequent analyses.

**Table 1:** the summary of GEO datasets involving UC and PsO.

Date Set	Control	Disease	Tissue	Group
GSE36807	7	15 UC	Colon	Training datasets
GSE47908	15	39 UC	Colon	Training datasets
GSE16879	12	24 UC	Colon	Verification datasets
GSE13355	122	58 PsO	Skin	Training datasets
GSE14905	49	33 PsO	Skin	Training datasets
GSE30999	85	85 PsO	Skin	Verification datasets

### 2.2 Differentially expressed genes analysis

Based on the preprocessed dataset, the R software limma package was used to conduct differential expression genes (DEGs) analysis for both the UC group versus the normal control group and the PsO group versus the normal control group. The criteria for screening differential genes were set as: adjusted *P* < 0.05 and | log_2_ fold change (log_2_FC) | >0.5. The distribution of DEGs was visualized using a volcano plot, where red represents significantly upregulated genes and blue represents significantly downregulated genes, and the number and expression patterns of upregulated and downregulated DEGs in UC and PsO (such as co-upregulation, co-downregulation, inverse regulation, etc.) were statistically analyzed.

### 2.3 Weighted gene co-expression network analysis

The R software “WGCNA” package was used to construct gene co-expression networks for UC and PsO. The optimal soft threshold (power value) was determined by analyzing scale independence and mean connectivity to ensure that the network meets the characteristics of a scale-free topology (fit index > 0.85). Gene clustering was executed utilizing the predetermined optimal soft thresholding power, thereby partitioning genes exhibiting correlated expression profiles into discrete co-expression modules. Module assignment and quantity were subsequently resolved via implementation of the dynamic tree cutting algorithm. Module-phenotype association analysis was used to filter key modules significantly associated with UC and PsO phenotypes (*P* < 0.05), and the results were visualized through cluster trees and module-trait relationship heatmaps.

### 2.4 Shared gene screening and functional enrichment analysis

We conducted an intersection analysis of the DEGs between UC and PsO, and further intersected these with key module genes selected through WGCNA, thus defining them as shared candidate genes for UC and PsO. The intersection results are visually presented using a Venn diagram. Functional enrichment analysis of the shared gene set was conducted using the **“**clusterProfiler**”** package within the R statistical computing environment, encompassing Gene Ontology (GO) annotations—spanning biological process, cellular component, and molecular function categories—as well as pathway enrichment analysis derived from the Kyoto Encyclopedia of Genes and Genomes (KEGG) database, using P<0.05 as the criterion for significant enrichment, and displayed the top enrichment items through a bubble chart. A protein–protein interaction (PPI) network comprising the identified shared hub genes was constructed utilizing the STRING database in conjunction with Cytoscape software.

### 2.5 Machine learning screening of diagnostic feature genes

Leveraging the shared candidate gene set, four distinct machine learning frameworks—namely, Support Vector Machine (SVM), Random Forest (RF), eXtreme Gradient Boosting (XGB), and Generalized Linear Model (GLM)—were deployed to identify diagnostic feature genes within the UC and PsO expression datasets. The intersection of the results from the four algorithms was taken as the core shared diagnostic genes for UC and PsO, with the intersection results displayed in a Venn diagram.

### 2.6 Diagnostic efficacy validation

The diagnostic performance of the core overlapping diagnostic genes was evaluated through both internal and external validation strategies. Internal validation utilized the training dataset, while external validation employed independent GEO datasets, from which only baseline (pretreatment) samples were retained: for UC, baseline samples from GSE16879; for PsO, baseline samples from GSE30999. Expression profiles of the core genes across distinct groups (normal control, UC, and PsO) were visualized using box plots. Diagnostic performance was subsequently assessed via Receiver Operating Characteristic (ROC) curve analysis, with the corresponding area under the curve (AUC) computed. An AUC value exceeding 0.7 was adopted as the threshold for denoting satisfactory diagnostic capacity.

### 2.7 Immune infiltration analysis

Immune cell composition within the UC and PsO expression datasets was characterized using the CIBERSORT algorithm to estimate the relative proportions of distinct immune cell subsets. The differences in immune cells between the control group and the disease group were compared using box plots and heat maps. Furthermore, associations between the identified feature genes and immune cell infiltration profiles were interrogated. Specifically, non-parametric Spearman rank correlation analysis was conducted to quantify the relationship between infiltrating immune cell subsets and the shared diagnostic genes.

### 2.8 Establishment of UC and PsO comorbidity animal model

Male C57BL/6 mice (7–8 weeks old, 20–22 g body weight) were obtained from Liaoning Changsheng Biotechnology Co., Ltd. (Liaoning, China). mice were permitted a one-week acclimatization period prior to experimental manipulation. All procedures involving animal subjects received approval from the Ethics Review Committee of Hubei University of Science and Technology (No. 2025-04-001). In this experiment, two disease control groups and disease model groups were established, with 6 mice in each group. The UC model group was induced with colitis using dextran sulfate sodium (DSS), specifically by administering a 3% DSS solution in drinking water for 7 consecutive days (26). In the PsO group, mice were induced to develop PsO by applying 5% imiquimod (IMQ) (Mingxin, Sichuan, China) to the shaved area on their backs for 8 consecutive days. The mice’s backs were shaved to a size of 2 cm× 2cm, and 62.5mg of IMQ was applied daily for 7 days. The combined model group received the same 3% DSS solution in drinking water while also applying an equal amount of 5% IMQ to the shaved area on their backs (27). This combined regimen constitutes an acute comorbidity model, in which UC and PsO are simultaneously induced to capture shared immediate inflammatory pathways, rather than to recapitulate the chronic sequential onset of the two diseases observed in clinical settings.

### 2.9 Morphological and histopathological detection

Colitis progression was monitored by serial weekly assessments of body weight fluctuation, fecal consistency, and hemorrhagic manifestation. The Disease Activity Index (DAI) was employed to quantify colitis severity according to the following scoring rubric: weight change (0 = no loss or gain; 1 = 5%–10% reduction; 2 = 11%–15% reduction; 3 = 16%–20% reduction; 4 = ≥21% reduction); stool consistency (0 = formed, normal pellets; 2 = loose, unformed stool; 3 = watery diarrhea); and fecal bleeding (0 = normal coloration; 2 = reddish stool; 3 = grossly bloody stool). Upon termination of the experimental period on day seven, the colon was excised from the anus to the cecum and its length measured. Colonic specimens were subsequently fixed in 4% phosphate-buffered saline (PBS)-buffered formaldehyde, embedded in paraffin, and sectioned at a thickness of 4 μm. Hematoxylin and eosin (H&E) staining was performed for histological evaluation. Histopathological scoring was conducted using the following scale: 0, absence of inflammatory signs; 1, mild inflammation characterized by scattered mononuclear infiltrates; 2, moderate inflammation with multifocal mononuclear aggregates; 3, marked inflammation accompanied by increased vascularization and pronounced mural thickening; 4, severe inflammation featuring transmural leukocytic infiltration and goblet cell depletion (28).

### 2.10 Real-time polymerase chain reaction (RT-qPCR)

To validate the expression profiles of shared diagnostic genes identified through bioinformatic analyses, total RNA was isolated from both colonic and cutaneous tissues using the FastPure Cell/Tissue Total RNA Extraction Kit V2 (Vinozan, China). Subsequently, 1 μg of total RNA was subjected to reverse transcription employing HiScript III RT SuperMix for qPCR (incorporating gDNA remover) to generate complementary DNA (cDNA). Quantitative real-time PCR was then performed on a real-time PCR detection platform utilizing Taq Pro Universal SYBR qPCR Master Mix with the synthesized cDNA serving as template. Primer sequences employed in this study are cataloged in **Table 2**, and all reaction assembly and thermal cycling parameters adhered strictly to the manufacturer’s prescribed protocols. Relative gene expression was quantified using the 2^^(–ΔΔCt)^ comparative threshold method.

**Table 2:** RT-qPCR primers information of the indicated DEGs.

Gene name	Forward primer (5’ to 3’)	Reverse primer (5’ to 3’)
CLDN8	GGAATGCCAATCCATCACGC	AGGCGTGTAGAGGGAAAGGA
ABCA12	CATCGGTGGCTCCAGGGTAG	CTTGGTGAGTGTGAGGTGGTAAC
GAPDH	GTGTTCCTACCCCCAATGTG	GTCATTGAGAGCAATGCCAG

### 2.11 Mendelian randomization

A bidirectional two-sample Mendelian randomization (MR) framework was implemented to rigorously evaluate the putative causal association between UC and PsO. To mitigate population stratification bias, all GWAS summary statistics were sourced from the IEU OpenGWAS database, exclusively restricting the analysis to individuals of European descent. For data integration, a total of 12 independent GWAS datasets were incorporated, comprising 6 datasets for UC and 6 for PsO (**Table 3**). By cross-pairing these datasets bidirectionally, we constructed 72 exposure-outcome combinations, including 36 pairs for the PsO-to-UC direction and 36 pairs for the UC-to-PsO direction, enabling a comprehensive exploration of causal directionality between the two diseases. Independent genetic variants significantly associated with the exposure factor were screened using a genome-wide significance threshold of *P* < 5×10^-8^, and associated single nucleotide polymorphisms (SNP) were removed using linkage disequilibrium clustering (r² < 0.001, cluster window = 10,000 kb). The F-statistic for each SNP was calculated, and weak instrumental variables with F < 10 were eliminated. For each exposure-outcome pair, the causal effect was estimated using inverse variance weighting, and heterogeneity and level pleiotropy were assessed using the Cochran’s Q test and the MR-Egger intercept test, respectively. To ensure robustness, only combinations simultaneously meeting the following criteria were interpreted: Number of SNPs (nsnp) ≥3, Minimum F-statistic (Min_F) >10, Heterogeneity Q-test (Het_Q_p) ≥0.05, and Pleiotropy test P (Egger_p) ≥0.05. All MR analyses were performed using the Two Sample MR package (version 0.5.8) in R software (4.1.2).

**Table 3:** Characteristics of the included GWAS datasets for UC and PsO.

GWAS ID	Trait	Population	Sample size
ebi-a-GCST90018907	PSO	European	483174
ebi-a-GCST90019016	PSO	European	44161
ebi-a-GCST90013885	PSO	European	407746
ebi-a-GCST90013935	PSO	European	407746
ukb-b-10537	PSO	European	462933
ukb-a-100	PSO	European	337159
ukb-a-553	UC	European	337199
ukb-a-104	UC	European	337159
ukb-b-19386	UC	European	463010
ukb-b-7584	UC	European	462933
ukb-b-7421	UC	European	463010
ukb-b-3044	UC	European	462933

### 2.12 Statistical analysis

All statistical analyses were performed using R software (version 4.3.2) and GraphPad Prism 9.0 software. Measurement data are presented as mean ± standard deviation (Mean ± SD), and comparisons between two groups were made using Student’s t-test. For differential genes, module correlation, and correlation analysis, the statistical significance criterion was set at *P* < 0.05. Data visualization was performed using the ggplot2 and “pheatmap” packages.

## 3 Results

### 3.1 Differential expression gene analysis

**Figure 2:**
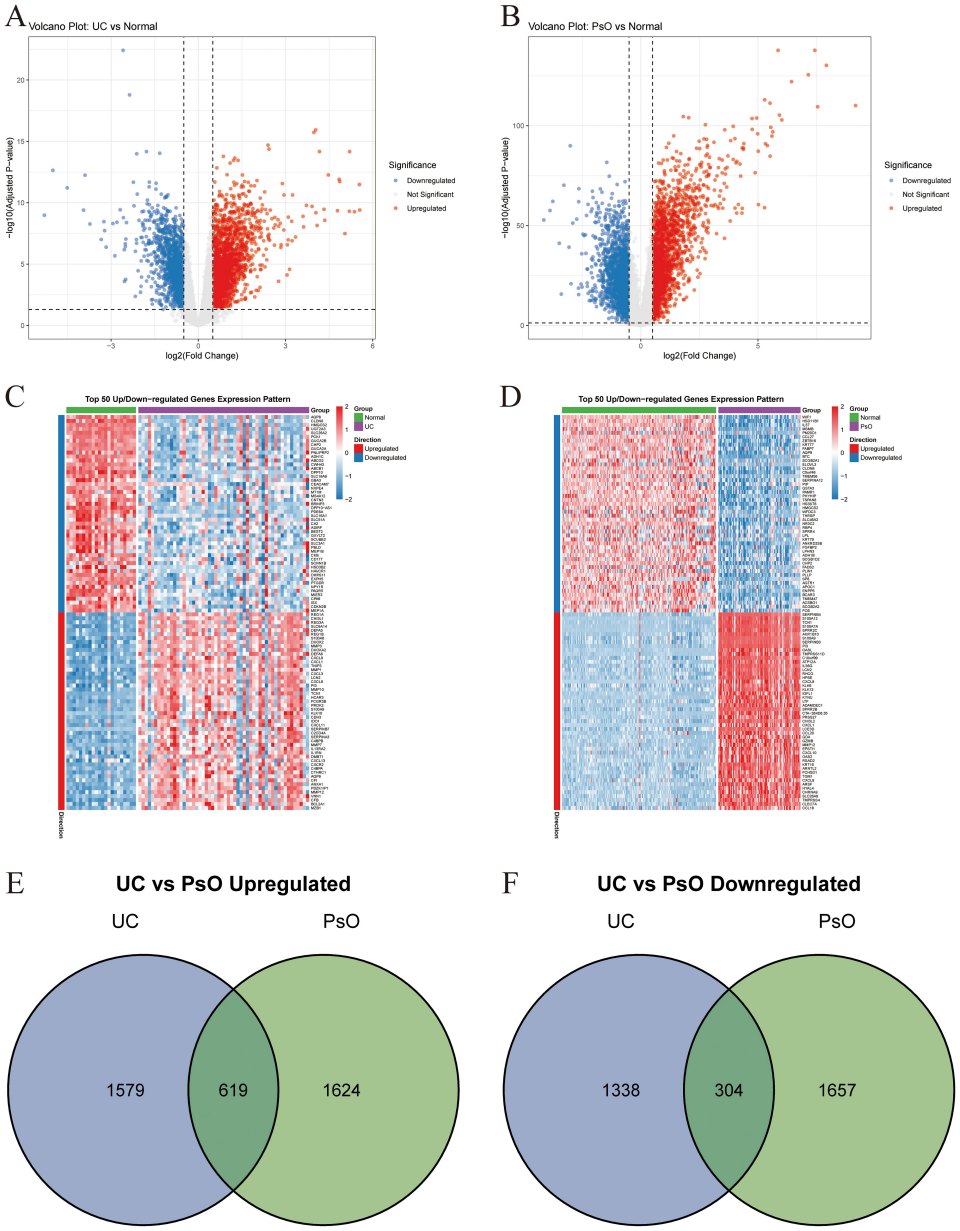
DEGs in UC and PsO screening. **(A, B)** Volcano plots for UC and PsO. **(C, D)** Heat maps for UC and PsO. **(E, F)** Venn diagram of upregulated and downregulated DEGs in UC and PsO.

To test the shared mechanism hypothesis, we first identify the differentially expressed genes and co-expression modules shared between UC and PsO. After the normalization of GSE36807 and GSE47908, 22 control and 54 UC colon samples were included in this study (**Supplementary Figure S1**). 3840 DEGs were identified in UC, including 2198 upregulated and 1642 downregulated genes (**Figure 2A**). PsO gene expression profile from GSE13355 and GSE14905 were standardized, involving 171control and 91 PsO samples. A total of 4208 DEGs in PsO were identified, with 2246 genes upregulated and 1962 genes downregulated (**Figure 2B**). To visualize the DEGs data, we used volcano plots, where red indicates highly expressed upregulated genes, while blue represents lowly expressed downregulated genes (**Figures 2C, D**). By intersecting those UC DEGs and PsO DEGs, it was found that there were 619 overlapping upregulated genes and 304 overlapping downregulated genes between UC and PsO (**Figures 2E, F**). These findings delineate the intricate transcriptional interplay linking UC and PsO and may further implicate disease-specific regulatory circuits operative within these chronic inflammatory conditions.

### 3.2 Identification of key module genes using WGCNA

**Figure 3:**
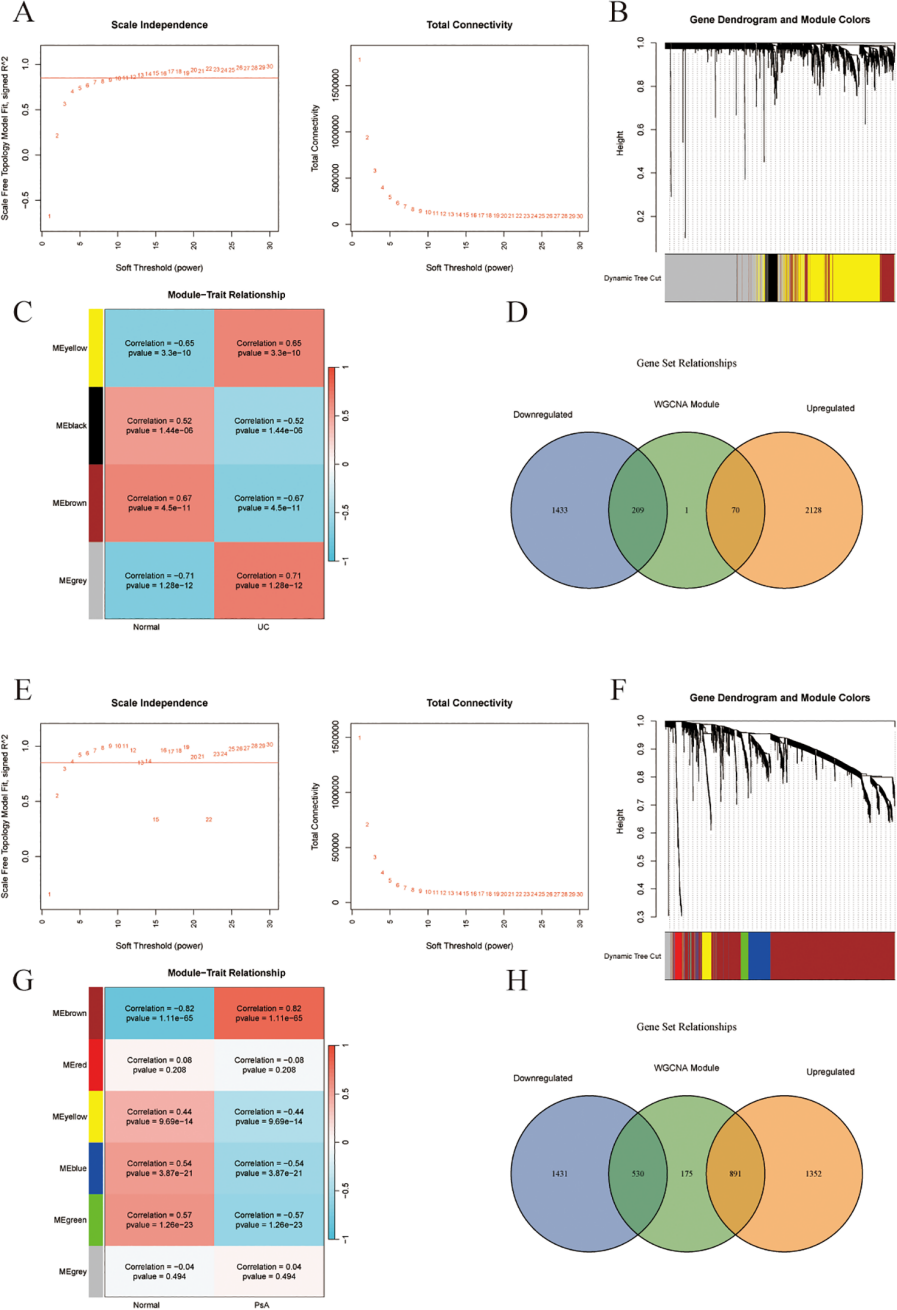
Module genes screening in UC and PsO by WGCNA. **(A)** Determination of soft threshold and clustering tree for the UC. **(B)** Correlation between modules and traits in the UC. **(C)** Determination of correlation between modules and traits in the UC. **(D)** Venn diagram of upregulated DEGs, downregulated DEGs and modules genes in UC. **(E)** Determination of soft threshold and cluster tree for the PsO. **(F)** Correlation between modules and traits in the PsO. **(G)** Correlation between modules and traits in the PsO group. **(H)** Venn diagram of upregulated DEGs, downregulated DEGs and modules genes in the PsO.

To complement the single-gene perspective from our differential expression analysis and to uncover higher-order functional relationships, we constructed a co-expression network using WGCNA. In the UC dataset, a soft thresholding power of 30 was selected to achieve a scale-free topology fit exceeding 0.85. Based on this parameter, a co-expression network was generated, culminating in module assignment and the derivation of corresponding clustering trees (**Figures 3A, B**). Four distinct co-expression modules were delineated in total. Of these, the brown module exhibited the most robust correlation with UC status (**Figure 3C**). From the UC-related brown module in WGCNA, which comprised 280 genes, a set of hub DEGs was derived by taking the intersection with the DEGs list. This resulted in the identification of 70 up-regulated and 209 down-regulated hub DEGs (**Figure 3D**). Subsequently, we also applied WGCNA to identify key modules in PsO. The PsO expression profile was segregated into five distinct co-expression modules (soft threshold =0.85; **Figures 3E, F**). Notably, the brown module again demonstrated the highest correlation with the disease. By taking the intersection between genes in the PsO brown module and the previously defined DEGs, we identified 891 up-regulated and 530 down-regulated hub DEGs (**Figures 3G, H**). Taken together, the hub DEGs identified through this convergent analysis in both UC and PsO provide a strong foundation for comparative analysis and further functional investigation into shared and unique pathogenic mechanisms.

### 3.3 Identification of shared hub genes and functional enrichment analysis

**Figure 4:**
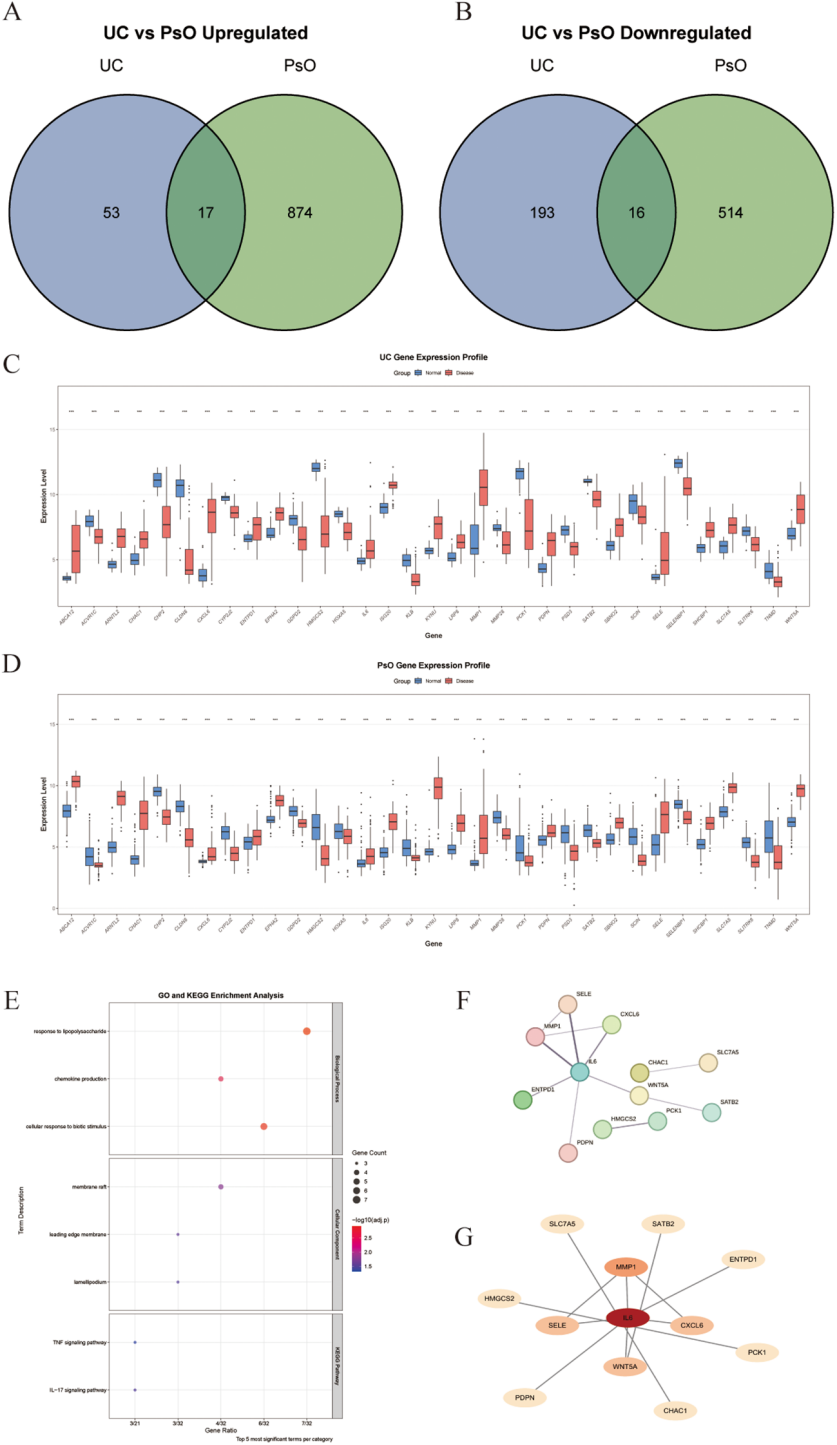
DEGs intersection of UC and PsO and its enrichment analysis. **(A, B)** Venn diagrams of upregulated and downregulated DEGs in UC and PsO, respectively. **(C, D)** The gene expression profiles of the 33 candidate genes selected from the training datasets in UC and PsO. **(E)** Visualization of enrichment analysis for UC and PsO. **(F, G)** PPI network diagram.

To further elucidate the core molecular drivers underlying the comorbidity of UC and PsO, we integrated the hub genes derived from WGCNA with the DEGs identified from each disease. Specifically, the 70 up-regulated UC hub DEGs were intersected with the 891 up-regulated PsO hub DEGs, revealing a robust set of 17 consistently up-regulated key genes common to both conditions (**Figure 4A**). Similarly, the intersection of the 209 down-regulated UC hub DEGs with the 530 down-regulated PsO hub DEGs yielded 16 consistently down-regulated key genes (**Figure 4B**). Therefore, we obtained 33 common genes for UC and PsO. Through gene expression analysis of these 33 common genes using the training set, as shown in the violin plots, there were significant changes in the expression of candidate genes in UC and PsO tissue lesions compared to healthy controls (**Figures 4C, D**). This rigorous cross-disease comparison pinpointed a total of 33 high-confidence candidate genes central to UC-PsO comorbidity. Subsequent GO and KEGG pathway enrichment analyses were performed on these 33 pivotal genes to decipher their collective biological implications. In the GO biological process analysis, the core genes were significantly enriched in the response to lipopolysaccharide (LPS), cellular response to biotic stimulus, and chemokine production, suggesting that a robust inflammatory response is central to the comorbidity and implicating gut microbiota-derived LPS in potentially activating shared immune mechanisms. For cellular components, the genes were notably localized to membrane rafts, the leading-edge membrane, and lamellipodium, highlighting the importance of immune cell (e.g., T cells, macrophages) migration and infiltration in the comorbid pathology. KEGG pathway analysis identified five significantly enriched pathways, including Lipid and atherosclerosis, IL-17 signaling pathway, PPAR signaling pathway, Rheumatoid arthritis, and TNF signaling pathway. Notably, the core genes were most prominently enriched in the TNF and IL-17 signaling pathways (**Figure 4E**), further underscoring the critical role of pro-inflammatory cytokine cascades in driving the synergistic pathogenesis of UC and PsO. To further elucidate the functional interplay among the 33 shared hub genes, a PPI network was constructed and analyzed. The resulting network revealed a significant degree of molecular connectivity, with several key inflammatory mediators and chemokines emerging as central nodes. Notably, IL-6, MMP1, and CXCL6 were identified as pivotal hubs within the network (**Figures 4F, G**), underscoring their dominant roles in the comorbid pathogenesis. These core proteins, primarily functioning as potent inflammatory cytokines and chemotactic factors, strongly suggest that the dysregulation of a cohesive immunoinflammatory module, centered around these factors, is a critical mechanism driving the synergy between UC and PsO. These findings compellingly suggest that the identified shared hub genes are critically involved in dysregulating core inflammatory circuits, potentially bridging the pathogenic mechanisms of UC and PsO at a molecular level. This integrated analysis not only highlights the innovative multi-modal bioinformatic strategy employed but also sharply focuses on the specific molecular ensemble—the 33 key genes—driving the shared immunopathology, thereby providing a solid foundation for the subsequent diagnostic model construction and experimental validation.

### 3.4 Identification of shared diagnostic biomarkers using machine learning algorithms

**Figure 5:**
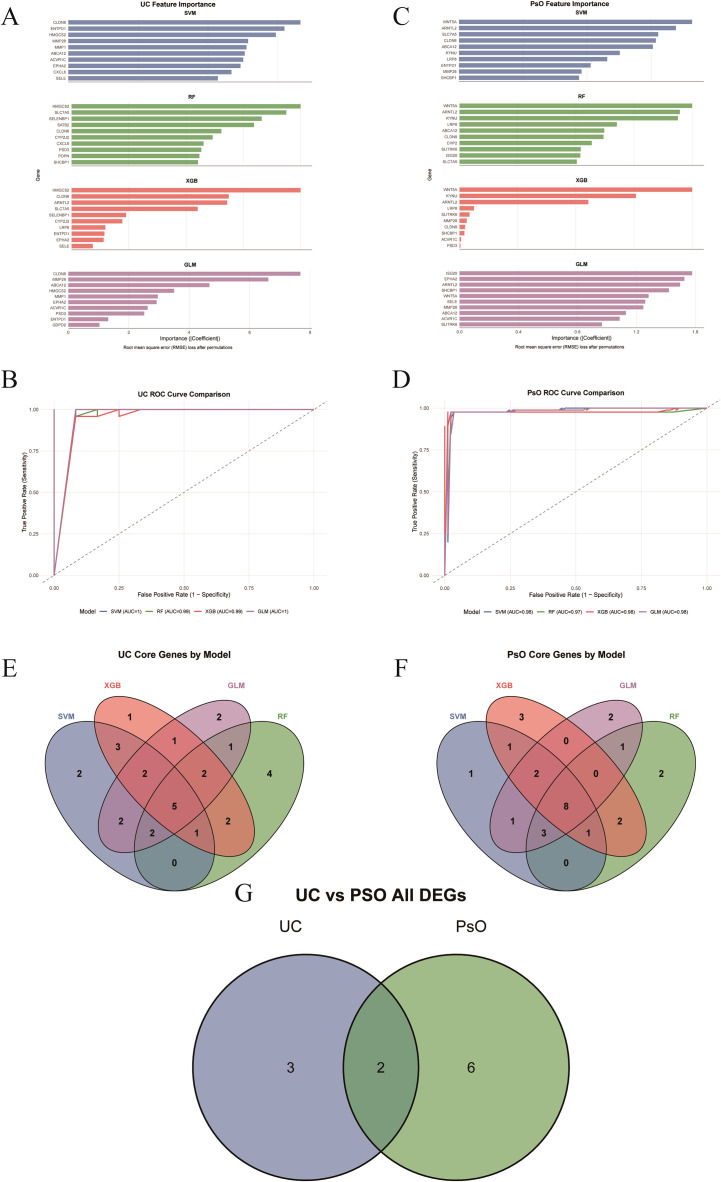
Screening shared genes for UC and PsO using four machine learning algorithms. **(A, C)** The training datasets for UC and PsO were screened using SVM, RF, XGB, and GLM machine learning methods respectively, displaying the top 10 genes for each machine learning method. **(B, D)** The genes selected for UC and PsO were subjected to ROC visualization analysis using the validation datasets GSE16879 and GSE30999 respectively. **(E, F)** Venn diagrams of the genes selected by the UC and PsO models. **(G)** Venn diagram of the intersection between UC and PsO.

To identify robust diagnostic biomarkers capable of distinguishing disease states from controls, we employed four distinct machine learning algorithms: SVM, RF, XGB, and GLM. The predictive power of these models was rigorously evaluated using ROC curves. For UC, the models demonstrated excellent diagnostic performance, with AUC values of 0.97 (SVM), 0.91 (RF), 0.92 (XGB), and 0.97 (GLM) (**Figures 5A, B**). Similarly, in PsO, all models achieved outstanding AUCs of 0.98 (SVM), 0.97 (RF), 0.98 (XGB), and 0.98 (GLM) (**Figures 5C, D**). Subsequently, the top 10 feature genes from each algorithm were intersected to pinpoint the most consistent and reliable biomarkers. This integrative approach identified a 5-gene overlap from the UC models and an 8-gene overlap from the PsO models. The final intersection between these two gene sets yielded two core shared diagnostic genes: CLDN8 and ABCA12 (**Figures 5E–G**). This multi-algorithmic strategy effectively narrows down the candidate pool and highlights CLDN8 and ABCA12 as paramount, high-confidence biomarkers for UC-PsO comorbidity.

### 3.5 Establishment of diagnostic prediction model

**Figure 6:**
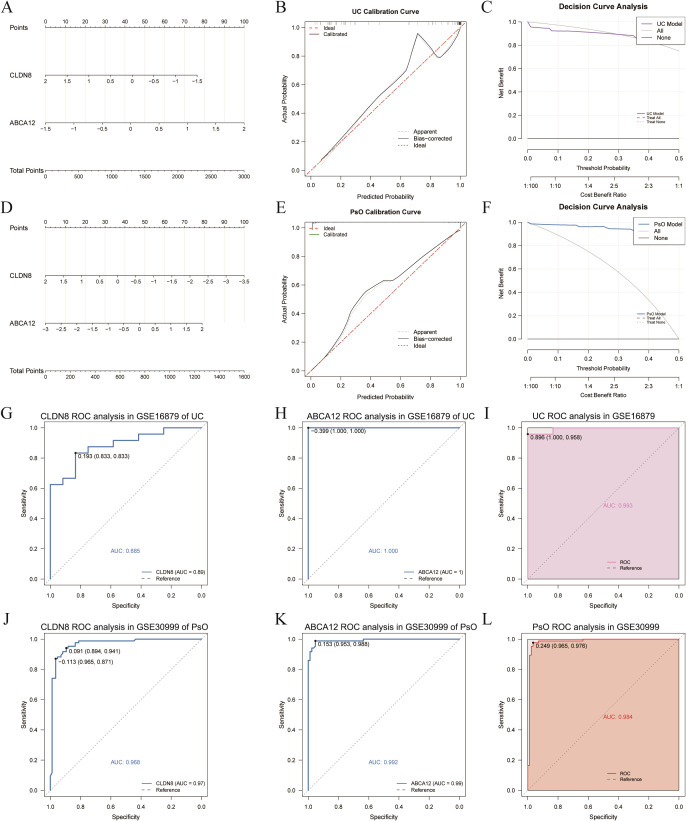
Nomogram model construction. **(A, D)** show the nomogram diagnostic models in UC and PsO. **(B, E)** shows the calibration curve graphs in UC and PsO. **(C, F)** show the decision curve analysis (DCA) plots in UC and PsO. **(G, H)** plotted ROC curve analyses for the CLDN8 and ABCA12 genes in the UC validation set (GSE16879). **(I)** Using the UC validation set (GSE16879) for the model's ROC curve. **(J, K)** plotted ROC curve analyses for the CLDN8 and ABCA12 genes in the PsO validation set (GSE30999). **(L)** Using the PsO validation set (GSE30999) for the model's ROC curve.

To further verify the clinical practicality of the model and the reliability of the molecular mechanisms, this study constructed a nomogram diagnostic model for PsO and UC and conducted multi-dimensional validation. In the UC model (**Figure 6A**), CLDN8 shows a strong negative contribution (-1.5 to 2 points), making it a dominant factor in UC risk; ABCA12 shows a weak positive contribution (-1.5 to 2 points), having a minor impact on disease risk. The total score ranges from 0 to 3000 points, with higher scores corresponding to a higher UC risk. For the PsO model (**Figure 6D**), the gene scoring point scale indicates that CLDN8 contributes negatively (-3.5 to 2 points), with lower scores correlating to higher disease risk; ABCA12 exhibits a bidirectional regulation (-3 to 2 points), where negative points reduce risk and positive points increase risk, with a total score range of 0 to 1600 points, where higher scores correspond to higher disease risk. Mechanistically, CLDN8, as a tight junction protein, plays a negative regulatory role in both diseases by maintaining epithelial barrier integrity, potentially serving as a core intervention target across diseases; ABCA12, as a lipid transport protein, has a greater weight in PsO, possibly closely related to abnormal skin lipid metabolism, while its role in UC is weaker, reflecting the tissue-specific differences in the pathological mechanisms between the gut and skin. Calibration curve analysis (**Figures 6B, E**) shows that the “Calibrated” curve closely aligns with the “Ideal” diagonal line, and the “Bias-corrected” curve, after bias correction, still maintains good consistency, indicating that the predicted probabilities of the UC and PsO models highly match the actual disease occurrence probabilities. The models exhibit excellent calibration, allowing precise quantification of individual disease risk and providing a reliable basis for early intervention in high-risk populations. Decision curve analysis (**Figures 6C, F**) further confirms that the UC model has significantly greater net benefits than the “Treat All/None” strategy within a broader threshold probability range of 1:10 to 1:1, offering wider clinical applicability and more flexible guidance for disease stratified prevention; the PsO model shows significantly higher net benefits than the “Test All/None” strategy within a threshold probability range of 1:4 to 2:3, providing the best intervention guidance for moderate-risk populations. In summary, the nomogram model constructed in this study has been validated through calibration curves and decision curves, demonstrating good predictive accuracy and clinical practicality. CLDN8, as a core target across diseases, provides a molecular basis for epithelial barrier repair strategies in the treatment of co-morbidity between PsO and UC; the higher weight of ABCA12 in PsO suggests that skin-specific lipid metabolism regulation may be a potential direction for its precise treatment. In clinical practice, the UC gene scoring model can be widely applied for population screening and stratified management, while the PsO model is more suitable for optimizing individualized interventions for moderate-risk patients, providing bioinformatics support and decision-making references for the precise diagnosis and treatment of inflammatory diseases. Comparative assessment of expression patterns and diagnostic performance across validation cohorts revealed that both CLDN8 and ABCA12 maintained expression trajectories consistent with those observed in the training dataset, suggesting their potential as candidate diagnostic indicators for UC and PsO. Collectively, these data provide preliminary support for the candidacy of CLDN8 and ABCA12 as candidate biomarkers that may aid in the molecular discrimination of these conditions; however, their clinical applicability remains to be validated in prospective clinical studies.

### 3.6 Analysis of immune infiltration involving the shared diagnostic gene

**Figure 7:**
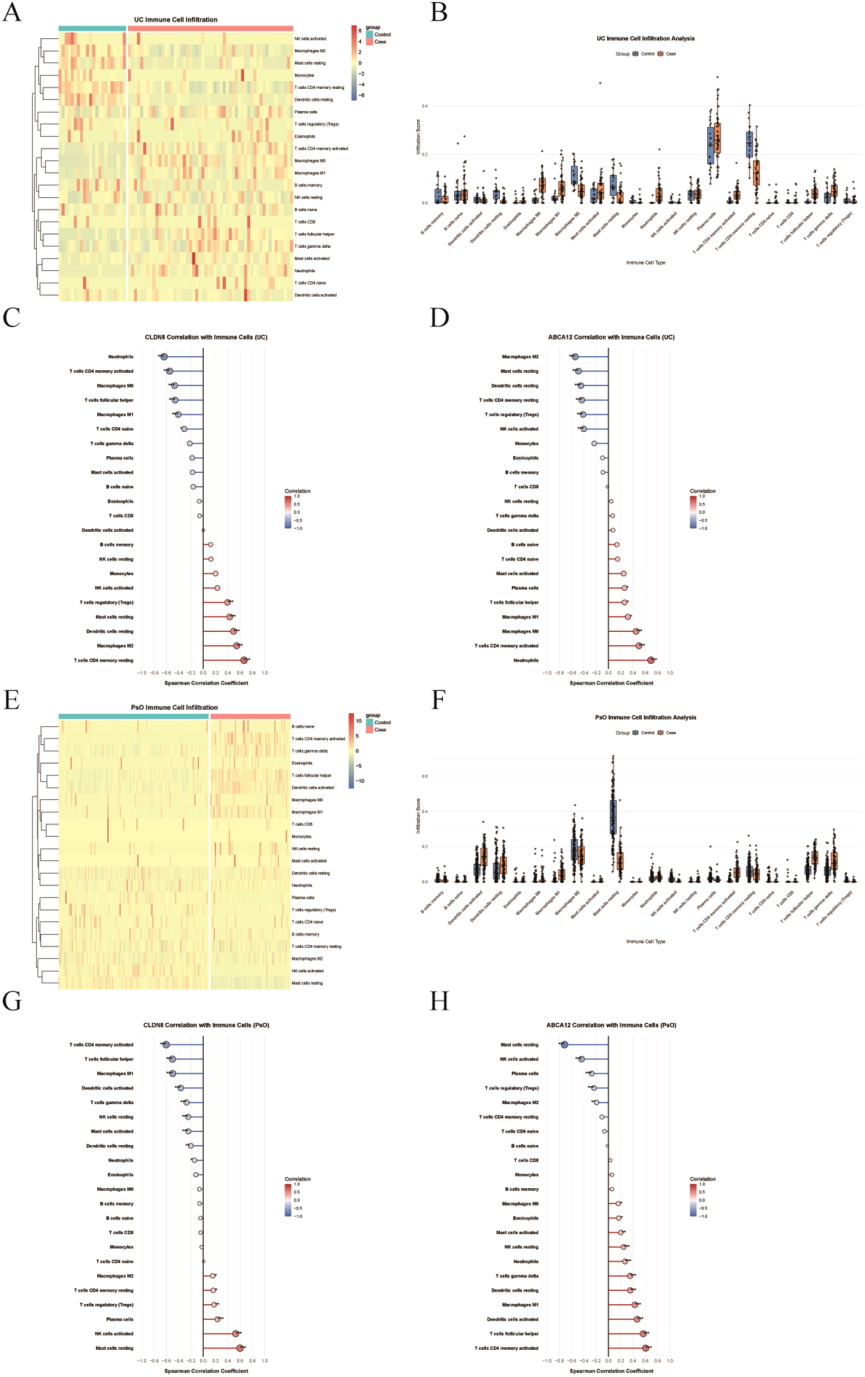
Immune infiltration analysis in both UC and PsO. **(A)** Heatmap of immune cells in UC. **(B)** Boxplot showing the differential composition of various types of immune cells between UC samples and control samples. **(C)** Correlation of CLDN8 expression with various immune cells in UC. **(D)** Correlation of ABCA12 expression with various immune cells in UC. **(E)** Heatmap of immune cells in PsO. **(F)** Boxplot showing the differential composition of various types of immune cells between PsO samples and control samples. **(G)** Correlation of CLDN8 expression with various immune cells in PsO. **(H)** Correlation of ABCA12 expression with various immune cells in PsO.

The results of the CIBERSORT analysis show that there are significant changes in the infiltration patterns of immune cells when comparing the UC group and the PsO group to the control group. In the immune microenvironment of UC and PsO, major inflammatory cells such as neutrophils, M1 macrophages, and activated CD4 memory T cells all show an increasing trend, while immunoregulatory cells such as M2 macrophages, resting dendritic cells, and resting CD4 memory T cells exhibit a decreasing trend (**Figures 7A, B, E, F**). It is particularly noteworthy that in the immune microenvironment of UC, neutrophils also demonstrate an upregulation, leading to a neutrophil-dominated acute inflammatory response, while resting dendritic cells show a downregulation. In the immune microenvironment of PsO, the number of γδT cells significantly increases. These two situations are interrelated, ultimately resulting in a significant polarization of Th1/Th17 immune responses, a severe imbalance in the M1/M2 macrophage ratio, enhanced activation status of natural killer (NK) cells, and dysregulation of key immune indicators related to epithelial barrier function. Correlation analysis further revealed a significant association between the expression levels of the CLDN8 and ABCA12 genes and the degree of infiltration of different immune cell subpopulations. Specifically, the CLDN8 gene is negatively correlated with neutrophils (r=-0.92, *P* < 0.001), M1 macrophages (r=-0.65, *P* < 0.01), and activated CD4 memory T cells (r=-0.91, *P* < 0.001), while showing a strong positive correlation with resting CD4 memory T cells (r=0.82, *P* < 0.001). The ABCA12 gene is positively correlated with M2 macrophages (r=0.85, *P* < 0.001) and resting mast cells (r=0.89, *P* < 0.001). At the same time, it shows significant negative correlations with neutrophils (r=-0.78, *P* < 0.001) and M1 macrophages (r=-0.72, *P* < 0.01) (**Figures 7C, D, G, H**). Immune infiltration analysis indicates that the CLDN8 and ABCA12 genes play a crucial role in the immune responses targeting T cells, macrophages, and dendritic cells in the pathogenesis of UC and PsO.

### 3.7 Establishment of a mouse model of UC and PsO comorbidity

**Figure 8:**
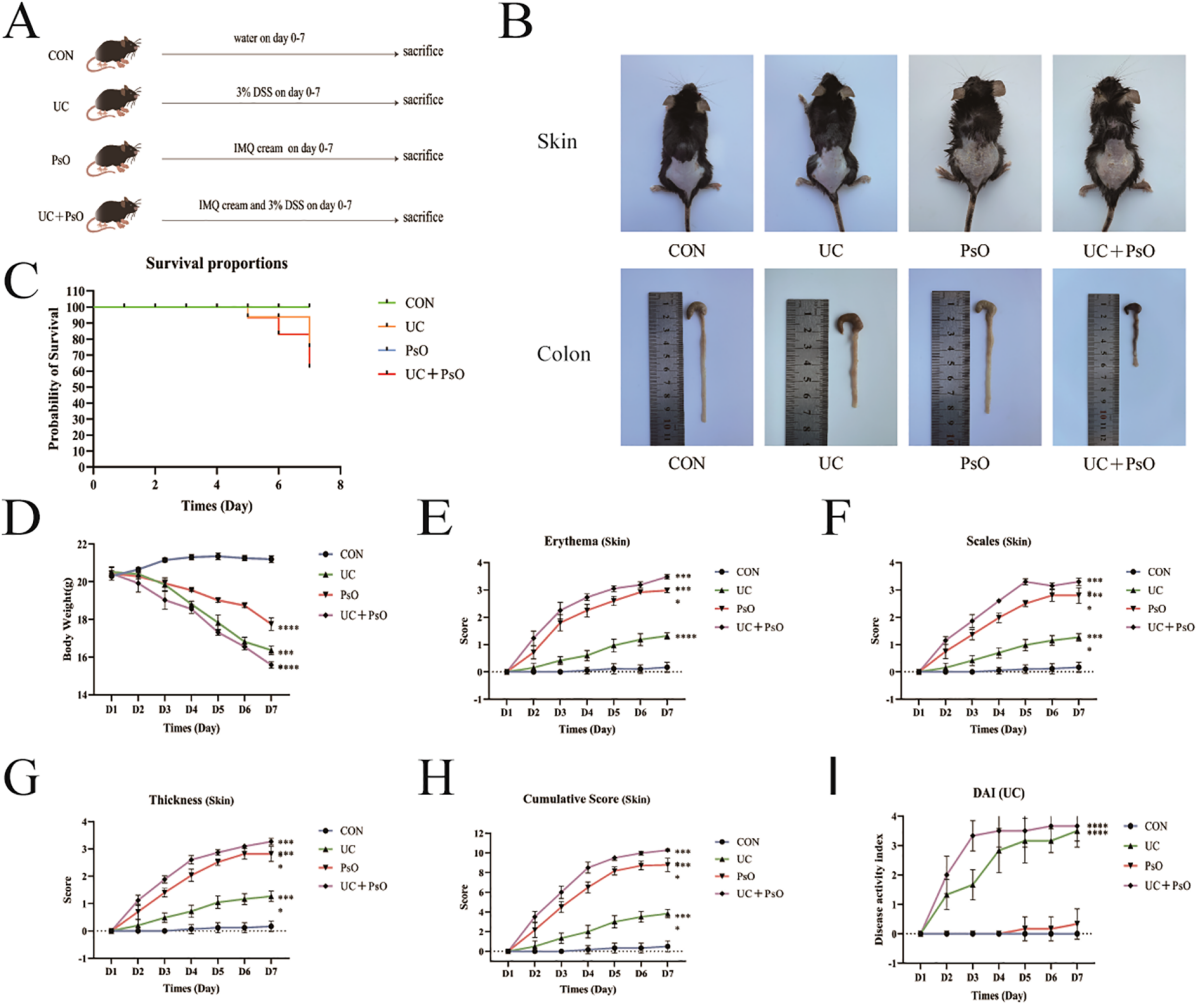
UC and PsO comorbidity mice model construction. **(A)** UC and PsO mice model construction, including healthy control (CON), DSS-induced UC group (UC), IMQ-induced PsO group (PsO), and DSS and IMQ combined group (UC+ PsO). **(B)** Skin appearance and colon length on the 7th day for the all groups. **(C)** Survival curve analysis of the four groups. **(D)** Weight changes during treatment in all groups. **(E–H)** Scoring of back skin erythema, scaling, and thickness daily on a scale of 0 to 4. Cumulative scores (erythema, scales, thickness) are described. Symbols indicate mean ± S.D. (n=1/45). **(I)** DAI changes in all groups. Statistical analysis was performed using Student's t-test. *p < .05, **p < .01, ***p < .001, **** *p* < 0.0001.

To verify the functional relevance of the shared genes mentioned above, a UC-PsO comorbidity animal model was constructed, we next assessed the severity of intestinal inflammation in colitis mice after applying imiquimod to induce PsO on their skin. A comorbidity model of PsO and UC in mice was established by selecting an appropriate dose of imiquimod and DSS concentration (**Figure 8A**). To validate the effectiveness of the animal model, we scored the DAI of the experimental mice (**Figure 8D**) and monitored their weight (**Figure 8I**), and the survival curve is shown (**Figure 8C**). Additionally, the skin lesions of PsO mice were scored using the PASI scale (**Figures 8E–H**). The results indicated that the mice in the combined model exhibited more severe inflammation than those with colitis alone, as evidenced by colonic length and histological scores (**Figure 8B**).

### 3.8 Histopathological features and inflammatory factor expression

**Figure 9:**
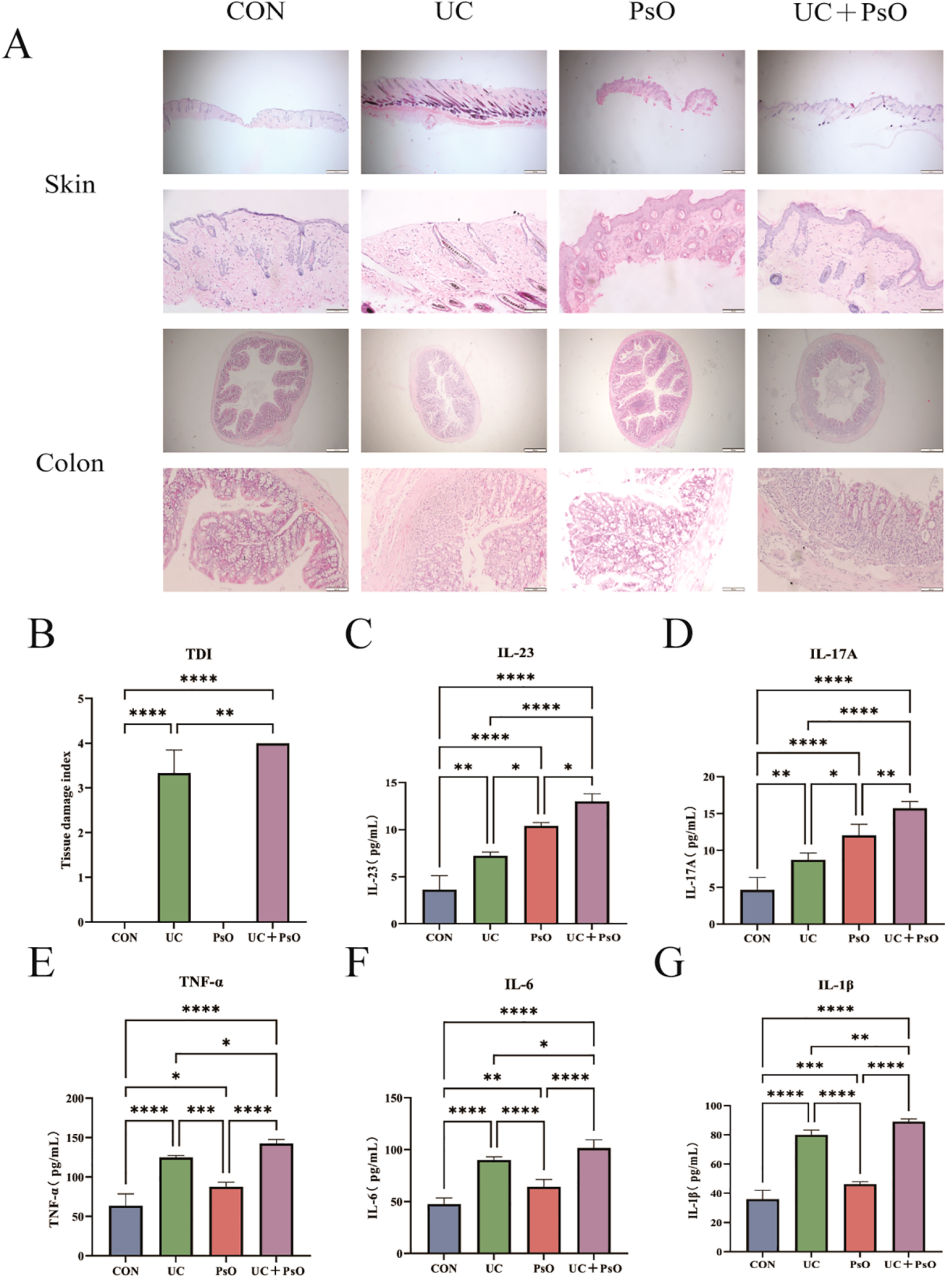
UC and PsO comorbidity exacerbate each other's tissue damage. **(A)** H&E staining of skin tissue and colon tissue (scale: 500μm, 100μm). **(B)** Colon TDI changes in all groups. **(C, D)** IL-23 and IL-17A levels in mouse serum by ELISA. **(E–G)** TNF-α, IL-6, and IL-1β levels in mouse serum by ELISA. Data are presented as mean ± SD, n = 6. * *p* < 0.05, ** *p* < 0.01, *** *p* < 0.001, **** *p* < 0.0001 compared to CON group.

Morphological assessment of colon and skin tissues by H&E staining (**Figure 9A**). Compared to the colitis-only group, the intestinal and skin tissues of the combined model group lost their normal tissue morphology, consistent with previous reports (29). In contrast, the colonic tissue damage in the UC group was less severe than that in the combined model group, although infiltrating inflammatory cells were still visible, and skin tissue damage was also milder. The colonic tissue damage in the PsO group was less severe, but skin tissue damage was more pronounced, second only to the combined model group (**Figure 9B**). Further detection of inflammatory gene expression found. Compared to the UC group, the levels of TNF-α, IL-6, and IL-1β were significantly elevated in the intestinal tissues of the combined model group mice (**Figures 9E–G**). At the same time, compared to the PsO group, IL-23 and IL-17A were also significantly increased (**Figures 9C, D**). TNF-α, IL-6, and IL-1β are all important pro-inflammatory cytokines that play a significant role in the occurrence and development of UC, while IL-23 and IL-17A are key inflammatory factors in PsO. These findings indicate that PsO can exacerbate colitis damage, leading to the secretion of more inflammatory factors.

### 3.9 Verification of CLDN8 and ABCA12 expression in UC and PsO mice by RT-qPCR

**Figure 10:**
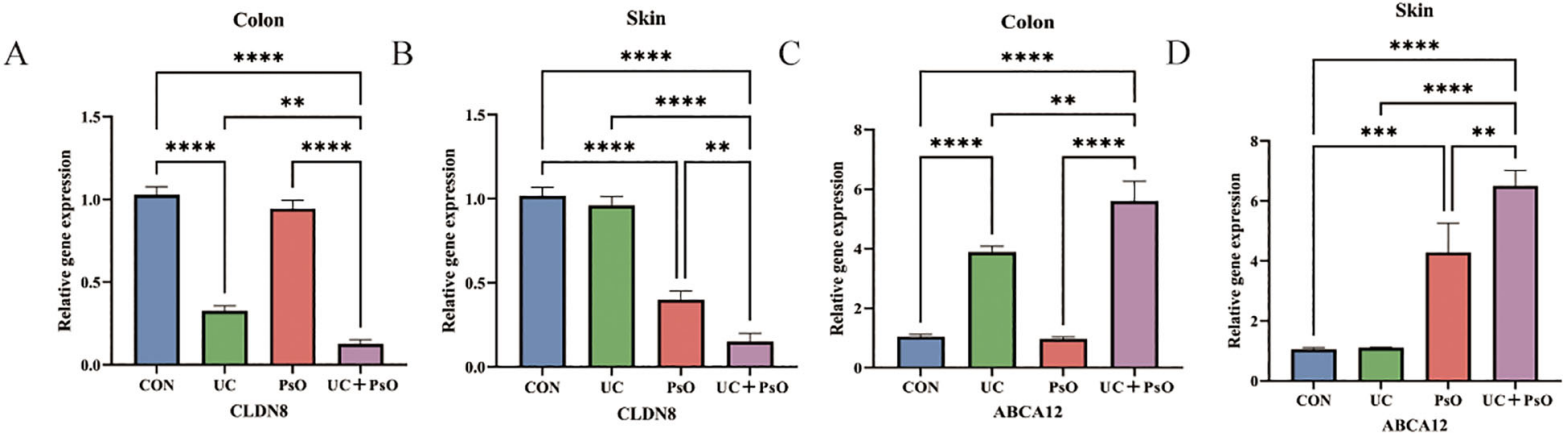
CLDN8 and ABCA12 expression levels in UC and PsO mice by RT-qPCR. **(A, B)** Expression levels changes of CLDN8 in colon and skin tissue. **(C, D)** Expression levels changes of ABCA12 in colon and skin tissue. * *p* < 0.05, ** *p* < 0.01, *** *p* < 0.001, **** *p* < 0.0001.

We conducted RT-qPCR experiments on the intestinal tissues of mice from the blank model, colitis model, and combined model, as well as on the skin tissues of mice from the blank model, PsO model, and combined model. The experimental results support the conclusions obtained from bioinformatics analysis, showing that CLDN8 is downregulated in the intestinal tissues of DSS-induced colitis mice, while also exhibiting downregulation in the skin tissues of imiquimod-induced PsO mice (**Figures 10A, B**). Additionally, ABCA12 is upregulated in the intestinal tissues of DSS-induced colitis mice, whereas it shows an upregulation trend in the skin tissues of imiquimod-induced PsO mice as well (**Figures 10C, D**).

### 3.10 Mendelian randomization reveals the causal relationship between UC and PsO

To distinguish between the direct causation hypothesis and the shared mechanism hypothesis, bidirectional MR was used to assess the genetic causal relationship between UC and PsO. If the MR results show no significant causal effect, it supports the shared mechanism hypothesis. This study employed a two-way, two-sample MR design, analyzing 72 exposure-outcome combinations based on multiple independent GWAS datasets (36 pairs in the PsO→UC direction and 36 pairs in the UC→PsO direction). Through rigorous quality control screening (nSNP ≥3, Min_F >10, Het_Q_p ≥0.05, Egger_p ≥0.05), 19 combinations were ultimately included for causal effect interpretation (**Table 4**). The two-way MR analysis results showed that in the PsO→UC direction, most of the selected combinations had negative effect estimates, but only the combination ukb-a-100→ukb-b-3044 reached marginal significance (*P* = 0.0112), while the remaining combinations were not statistically significant. In the UC→PsO direction, although the selected combination effect estimates showed some fluctuation, all *P >*0.05 and involved continuous traits; no statistically significant causal signal was observed. Based on the combined results of two-dimensional MR, this study did not find a consistent and statistically supported causal association between UC and PsO, suggesting that the comorbidity of the two diseases may mainly stem from shared genetic backgrounds or immunopathological mechanisms, rather than direct causal drivers.

**Table 4:** Bidirectional MR Analysis of UC and PsO after robustness filtering.

Direction	Exposure_ID	Outcome_ID	nSNP	Min_F	IVW_beta	IVW_p	Het_Q_p	Egger_p
UC -> PSO	ukb-a-104	ebi-a-GCST90018907	7	30.62	12.728	0.186	0.074	0.973
UC -> PSO	ukb-a-104	ebi-a-GCST90013935	7	30.62	13.160	0.593	0.206	0.099
UC -> PSO	ukb-a-104	ebi-a-GCST90013885	7	30.62	13.110	0.593	0.210	0.099
UC -> PSO	ukb-a-553	ukb-a-100	3	35.80	-0.150	0.220	0.437	0.623
UC -> PSO	ukb-a-553	ebi-a-GCST90018907	3	35.80	3.786	0.769	0.27	0.356
UC -> PSO	ukb-a-553	ebi-a-GCST90013935	3	35.80	52.194	0.114	0.742	0.583
UC -> PSO	ukb-a-553	ebi-a-GCST90013885	3	35.80	52.194	0.114	0.742	0.583
UC -> PSO	ukb-b-7584	ebi-a-GCST90018907	9	31.75	7.360	0.408	0.074	0.082
UC -> PSO	ukb-b-7584	ebi-a-GCST90013935	9	31.75	19.752	0.507	0.083	0.651
UC -> PSO	ukb-b-7584	ebi-a-GCST90013885	9	31.75	19.744	0.504	0.086	0.651
UC -> PSO	ukb-b-19386	ebi-a-GCST90013935	6	30.20	23.816	0.486	0.306	0.199
UC -> PSO	ukb-b-19386	ebi-a-GCST90013885	6	30.20	23.816	0.486	0.306	0.199
PSO -> UC	ukb-a-100	ukb-b-3044	9	29.99	-0.047	0.0112	0.832	0.901
PSO -> UC	ukb-a-100	ukb-b-7421	9	29.99	-0.006	0.765	0.713	0.313
PSO -> UC	ukb-b-10537	ukb-b-3044	9	31.03	-0.009	0.634	0.146	0.055
PSO -> UC	ukb-b-10537	ukb-b-7421	11	31.03	-0.023	0.216	0.118	0.468
PSO -> UC	ebi-a-GCST90018907	ukb-a-553	10	31.82	-0.000	0.803	0.062	0.466
PSO -> UC	ebi-a-GCST90018907	ukb-b-7421	4	31.82	-0.000	0.205	0.263	0.205
PSO -> UC	ebi-a-GCST90019016	ukb-b-7421	27	30.58	-0.000	0.519	0.079	0.067

Columns indicate the direction of analysis (exposure to outcome), Exposure Dataset ID,Outcome Dataset ID,number of instrumental SNPs (nsnp), minimum F-statistic (Min_F), inverse-variance weighted (IVW) beta estimate (IVW_beta) and its p-value (IVW_p), heterogeneity p-value (Het_Q_p), and MR-Egger intercept p-value (Egger_p) assessing horizontal pleiotropy. All exposure-outcome pairs shown passed stringent MR quality control (nsnp ≥3, Min_F >10, Het_Q_p≥0.05, Egger_p≥0.05).

## 4 Discussion

UC is a disease characterized by chronic non-specific inflammation that primarily affects the intestines, significantly impacting the quality of life of patients. Research indicates that the pathogenesis of UC is closely related to genetic susceptibility, environmental factors, and dysfunction of the immune system. With the rising incidence, UC has become an important public health issue globally, urgently requiring a deeper understanding to guide clinical treatment. At the same time, when UC coexists with other diseases, it can significantly worsen the patient’s condition. In clinical practice, the combination of UC and PsO not only increases the complexity of the disease but also affects the choice of treatment strategies, as there is a connection between the gut-skin axis in these two diseases, which may exacerbate each other’s symptoms. Although existing treatment options include medications and biological agents, their efficacy is often unsatisfactory, and side effects are prominent. This study aims to systematically investigate the correlation between UC and PsO-related comorbidities, utilizing gene expression data analysis to reveal the potential mechanisms of the two diseases. By applying various methods, including differential expression analysis, WGCNA, and machine learning, this study identified shared diagnostic genes for UC and PsO. These findings provide new insights into understanding the interactions between the two diseases and lay the groundwork for future treatment strategy development. The following discussion will focus on the main research findings, analyzing the identified common genes and their potential in clinical applications. Regarding the overlap of DEGs between UC and PsO, this study found a significant intersection in both upregulated and downregulated genes, suggesting that the two diseases may share certain molecular pathways (30). Previous studies have shown that both UC and PsO involve abnormal expression of immune-related genes and activation of immune pathways, particularly in aspects such as inflammatory responses, cytokine regulation, and barrier dysfunction (31). In our analysis, upregulated genes were predominantly enriched in immune activation, interferon signaling, chemokine secretion, and leukocyte recruitment pathways, with a series of chemokines (e.g., CXCL9, CXCL10, CXCL11, CCL20) and molecules mediating leukocyte migration and tissue infiltration significantly upregulated in both UC and PsO lesional tissues (32). Conversely, downregulated genes mainly focused on epithelial barrier maintenance, ion transport, and metabolic regulation, which aligns with previous reports that dysfunction of the intestinal mucosal and skin barrier is a common pathological basis of the two diseases (33). Notably, although the DEGs between the two diseases show high overlap, the activation intensity of related pathways and their manifestations in clinical phenotypes are not entirely identical, which may be related to multiple regulatory factors such as tissue microenvironment, epigenetic modifications, and microbiome interventions (34). Therefore, the overlap of DEGs between the two diseases provides a solid molecular foundation for revealing the comorbidity mechanisms, but also highlights the complexity of their specific regulatory networks (35). Furthermore, WGCNA analysis deepened our understanding of gene regulatory networks. We found 17 common upregulated and 16 common downregulated core gene modules in UC and PsO, which were predominantly related to immune signal transduction, inflammatory metabolic pathways, and tissue remodeling. Comparative studies have shown that WGCNA effectively reveals co-expression relationships between disease-related gene modules and captures key regulatory nodes that traditional differential analysis may overlook (36). The common genes identified in this study include adhesion molecules, tight junction proteins, and inflammatory mediators, which play a central role in immune-mediated chronic inflammation (37). Unlike previous studies focusing on single disease gene networks, we revealed a cross-tissue and cross-pathway molecular interaction pattern under the comorbid state of the two diseases through WGCNA, suggesting that signaling axes such as NF-κB and JAK-STAT may play bridging roles (38). In addition, in the lesional tissues of UC and PsO, abnormal activation of interferon signaling pathways, IL-23, and Th17-related pathways is associated with the abnormal expression of CLDN8 and ABCA12; these changes collectively drive the recruitment and activation of inflammatory cells, thereby triggering and sustaining a chronic inflammatory state (39, 40). However, this study did not find that certain classical autophagy or oxidative stress pathways dominated in the comorbidity modules of the two diseases, indicating that the comorbidity mechanism network leans more towards a model of immune inflammation and barrier damage synergistic regulation. In summary, the WGCNA results not only complement the deficiencies of differential expression analysis but also provide a theoretical basis for subsequent targeted interventions in the comorbidity network.Through the comprehensive application of various machine learning methods, this study further screened CLDN8 and ABCA12 as shared diagnostic genes for the two diseases. Compared to traditional single-algorithm screening, the integration of models such as SVM, RF, XGB, and GLM effectively enhanced the robustness and generalization ability of the results (41, 42). Previous explorations in related fields have mainly focused on biomarker discovery for single diseases, while this study overcame the errors caused by sample heterogeneity and feature redundancy through a multi-model ensemble strategy, significantly enhancing the clinical translatability of the intersecting genes (43). It is noteworthy that CLDN8 and ABCA12 have clear biological roles in barrier function, lipid metabolism, and immune regulation, having been confirmed to participate in tight junction structure maintenance and epithelial differentiation disorders, resonating with past findings regarding the core role of mucosal and skin barrier damage in UC and PsO. Additionally, reports on the joint machine learning screening of comorbidity diagnostic markers are extremely limited; this study achieved methodological breakthroughs, effectively expanding the application boundaries of bioinformatics in the research of multi-disease comorbidity mechanisms. Consequently, this study provides a new paradigm for the efficient identification of comorbidity genes and their clinical diagnostic value assessment.

The application of ROC curves in assessing the diagnostic potential of shared genes in this study indicated that CLDN8 and ABCA12 may possess favorable sensitivity and specificity for distinguishing UC and PsO from control samples. While ROC analysis has traditionally been applied to single biomarkers or clinical models, its use has been extended in recent years to evaluate the classification performance of multidimensional omics features and multiple disease states (44). Our results showed that, compared with single indicators, the combined use of candidate genes identified through machine learning yielded improved AUC values, and demonstrated consistency and generalization ability across independent datasets, which is in line with existing reports on the improvement of diagnostic accuracy via multi-biomarker joint models (45). It should be noted, however, that interpretation of ROC curves and AUC metrics in unbalanced samples or complex disease conditions requires careful consideration of specific thresholds and classification contexts (46). In the present study, repeated validation across multiple datasets and algorithms was performed to strengthen the rigor of the evaluation. Taken together, these ROC analyses provide preliminary support for the potential utility of CLDN8 and ABCA12 as candidate diagnostic biomarkers, yet their clinical applicability remains to be substantiated by well-designed prospective clinical studies before any consideration of diagnostic implementation. Animal model validation results further supported the functional role of CLDN8 and ABCA12 in the comorbid state of inflammatory bowel disease and PsO at the *in vivo* level. Several studies have confirmed that animal models play an irreplaceable role in revealing the pathological mechanisms of complex human diseases and validating key molecular targets, especially for comorbidity mechanism studies involving multiple systems and chronic inflammation (47). The comorbidity model constructed in this study not only replicated the human comorbid phenotype at the tissue pathology and molecular expression levels but also revealed a direct association between abnormal expression of CLDN8 and ABCA12 with barrier structural disorder and enhanced immune cell infiltration, consistent with previous findings regarding the central role of barrier destruction and local immune imbalance in the pathogenesis of UC and PsO (48). It is noteworthy that previous animal experiments often focused on single diseases or single gene functions; this study, through multi-molecular collaborative validation in comorbidity animal models, overcame the limitations of insufficient extrapolation of single disease models and promoted the transition of preclinical mechanistic research towards multi-system comprehensive interventions. Totally, animal model experiments provide strong pathological and molecular evidence for the key genes of comorbidity revealed in this study.

By integrating multidimensional bioinformatics analysis and bidirectional Mendelian randomization (MR), this study reveals two distinct features of the comorbidity between UC and PsO. On one hand, we identified and validated CLDN8 and ABCA12 as shared core diagnostic genes for both diseases, suggesting overlapping pathogenic mechanisms. On the other hand, MR analysis did not support a direct genetic causal relationship between UC and PsO. Collectively, these findings indicate that the comorbidity of UC and PsO mainly arises from genetic pleiotropy — that is, the same genetic loci or shared immune pathways can independently increase the risk of developing multiple diseases, but such associations do not equate to direct causal drivers between the diseases. CLDN8 and ABCA12 may serve as key mediators of the “gut−skin axis”, with their aberrant expression simultaneously disrupting intestinal epithelial barrier function and keratinocyte differentiation in the skin, thereby constituting a common molecular basis for the comorbidity (49). However, whether the final clinical manifestation is UC or PsO likely depends on differences in local tissue microenvironments, gut microbiota composition, and other environmental exposures, rather than one disease directly triggering the other. Unlike previous MR studies, the present study used a strictly defined UC (excluding Crohn’s disease cases) and more stringent criteria for instrumental variable selection, and detected no significant causal relationship (49). This discrepancy may be attributable to differences in disease subtype definition, population background, or instrumental variable selection. The limitations of this study mainly lie in sample selection and validation methods: the relatively small sample size may reduce statistical power and limit a comprehensive understanding of gene expression and disease mechanisms. In addition, batch effects across different datasets may affect the generalizability and applicability of the findings to other populations or clinical settings. In clinical practice, the existence of shared inflammatory pathways suggests that drugs targeting these pathways (e.g., IL−23 inhibitors) may theoretically provide therapeutic benefits for both diseases. Nevertheless, given the absence of a direct causal relationship between UC and PsO, clinicians should not assume that remission of one disease will automatically lead to improvement of the other. Instead, both conditions require independent and dynamic monitoring as well as targeted interventions. In summary, this study elucidates the intrinsic nature of UC−PsO comorbidity — shared specific molecular mechanisms (e.g., CLDN8− and ABCA12−related epithelial regulation) but no direct causal link — and provides candidate molecules and a theoretical basis for future functional studies and precision medicine investigations.

In the present study, our RT-qPCR results revealed that CLDN8 was consistently downregulated in both the colon and skin tissues of the experimental comorbidity models, whereas ABCA12 exhibited a modest but noticeable upregulation, particularly in the skin. The reduction of CLDN8 aligns with its established role as a tight junction component and is consistent with the impaired epithelial barrier function characteristic of both colitis and psoriasiform dermatitis. In contrast, the observed upward trend of ABCA12 in the IMQ−induced PsO model is unexpected, given that ABCA12 is canonically reported to be downregulated in psoriatic lesions as a result of Th1/Th17−driven disruption of keratinocyte differentiation. This apparent discrepancy prompted us to carefully re−examine the literature and consider possible explanations. Acute barrier disruption is known to trigger a compensatory barrier repair program in keratinocytes, during which pre−stored IL−1α is rapidly released and the unfolded protein response, together with nuclear receptors such as PPARγ, PPARβ/δ and LXR, are activated, leading to a transient upregulation of ABCA12 mRNA as part of the recovery process (50, 51). It is conceivable that, at the specific disease stage captured in our acute model, this reparative response temporarily outweighed the inhibitory signals from aberrant differentiation. A substrate−driven, feed−forward mechanism may also contribute to this pattern. ABCA12 expression is positively regulated by its own lipid substrates, particularly ceramides, which can activate PPARδ and subsequently stimulate ABCA12 transcription in a dose− and time−dependent manner (52). In the profoundly disturbed lipid metabolism of psoriatic epidermis, the local accumulation of certain long−chain ceramides may act as a “substrate signal” to drive ABCA12 upregulation.

Several limitations must be acknowledged when interpreting these findings. This study relates to the lack of protein-level validation and tissue localization of CLDN8 and ABCA12 in either animal models or clinical specimens. Therefore, the functional significance of this ABCA12 upregulation—whether it represents a meaningful compensatory response or merely a transient transcriptional fluctuation—remains to be determined by future studies using protein analysis, chronic models, or, ideally, tissue specimens from patients with concurrent UC and PsO. Ideally, immunohistochemical or immunofluorescence analyses in colon and skin tissues from acute and chronic comorbidity models would provide essential spatial information on how dysregulation of these molecules affects epithelial barrier integrity and immune cell infiltration. However, such experiments are currently constrained by practical obstacles. CLDN8 and ABCA12 are relatively niche targets, and the commercially available antibodies against these proteins have been developed and validated almost exclusively for human tissue specimens. To date, no antibodies have been rigorously validated for immunohistochemistry in mouse tissues, and applying human-specific antibodies to animal sections carries a considerable risk of nonspecific binding or false-negative results. In addition, the most clinically relevant validation would require matched colon and skin biopsies from patients with confirmed concurrent UC and PsO. Such comorbid specimens are exceedingly rare, and their collection is further hindered by stringent ethical requirements and logistical challenges. These combined constraints have prevented us from performing tissue-level validation within the scope of the present study. Consequently, the functional implications of CLDN8 and ABCA12 dysregulation on barrier function and immune responses remain speculative, and their verification must await the development of cross-reactive antibodies or the availability of comorbid patient specimens under appropriate ethical approval.

Notably, the findings presented are derived primarily from bioinformatics analyses of publicly available transcriptomic datasets, supplemented by limited experimental validation; therefore, the identified associations represent hypothesis-generating observations rather than clinically actionable findings. In particular, the proposed roles of CLDN8 and ABCA12 as candidate diagnostic biomarkers, as well as any suggestions regarding clinical decision-making, await independent validation in prospective clinical cohorts of patients with concurrent UC and PsO. In addition, the molecular crosstalk inferred from shared pathway enrichment does not establish causality, and functional studies are required to clarify whether the dysregulation of these genes actively contributes to comorbidity or merely reflects parallel inflammatory processes. Furthermore, the acute simultaneous DSS and imiquimod model was designed to capture shared acute inflammatory crosstalk, but it does not recapitulate the clinical temporal sequence—the mean interval between IBD and PsO diagnoses is approximately 5.4 years, and PsO incidence is higher after UC diagnosis in patients under 30. Thus, this single-phase acute model cannot reflect the sequential onset or chronic progression of the two diseases. Future work should employ chronic sequential models, such as chronic DSS-induced UC followed by imiquimod-induced PsO or vice versa, to validate the identified candidate molecules in a more clinically relevant setting. Moreover, heterogeneity of the datasets, differences in tissue sampling, and the lack of a dedicated comorbidity cohort may introduce confounding that limits the generalizability of our conclusions.

In addition, the total sample size of the datasets that met our stringent inclusion criteria, although pooled to a moderate scale, remains relatively limited. This may restrict the statistical power for detecting subtle transcriptomic differences and constrain the generalizability of the identified candidate biomarkers. As more high-quality transcriptomic datasets from UC and PsO patients become available in public repositories such as GEO and ArrayExpress, future studies should incorporate these additional cohorts to expand the validation scope and strengthen the external validity of the conclusions. Prospective clinical studies with larger sample sizes will also be needed to evaluate the diagnostic and prognostic utility of CLDN8 and ABCA12 in real-world settings.

## 5 Conclusion

In summary, this study identified CLDN8 and ABCA12 as potential candidate diagnostic genes for UC-PsO comorbidity, revealing shared molecular features involving epithelial barrier function and keratinocyte differentiation. This finding suggests that the comorbidity of these two diseases may share a common molecular basis. In clinical practice, these genes could potentially serve as candidate biomarkers for comorbidity risk screening and provide new directions for exploring therapeutic strategies targeting common inflammatory pathways. Although drugs targeting shared pathways (such as IL-23 inhibitors) may confer therapeutic benefits for both diseases simultaneously, given the distinct pathogenic bases of UC and PsO, clinical monitoring and intervention still need to be conducted separately, and this notion awaits validation by clinical studies. This study offers new insights into the molecular mechanisms of UC-PsO comorbidity and suggests that CLDN8 and ABCA12 are potential targets or candidate molecules worthy of attention in future functional research and precision medicine exploration.

## Data Availability

The raw data supporting the conclusions of this article will be made available by the authors, without undue reservation.
