# Supplementary material for: CLDN8 and ABCA12 define a shared molecular signature in ulcerative colitis-psoriasis comorbidity
Source: Front Immunol. 2026 Aug 5;17:1862149. doi: 10.3389/fimmu.2026.1862149 (PMC13485778; doi:10.3389/fimmu.2026.1862149)
Supplement: Supplementary file 1 [file SupplementaryFile1.docx]

**Supplementary Figure 1. Data preprocessing.** (A, B) Principal component analysis (PCA) plots of three UC datasets (GSE36807, GSE47908, and GSE16879) before (A) and after (B) normalization and batch correction, (C, D) PCA plots of three PsO datasets (GSE13355, GSE14905, and GSE30999) before (C) and after (D) normalization and batch correction.
